# Reduction of adhesion formation after knee surgery in a rat model by botulinum toxin A

**DOI:** 10.1042/BSR20160460

**Published:** 2017-04-20

**Authors:** Zheng-Yu Gao, Ji-Xia Wu, Wei-Bo Liu, Jin-Ke Sun

**Affiliations:** 1 Department of Rehabilitation, Affiliated Hospital of Qingdao University, Qingdao, Shandong 266003, China; 2 Department of Obstetrics, Affiliated Hospital of Qingdao University, Qingdao, Shandong 266003, China; 3 Department of Spine Surgery, Qingdao Center Hospital, Qingdao, Shandong 266042, China; 4 Department of Orthopaedics, Wendeng Orthopaedic Hospital of Shandong Province, Wendeng, Shandong 264400, China

**Keywords:** adhesion formation, botulinum toxin A, knee surgery

## Abstract

Adhesion of the knee is a major concern after knee surgery, the treatment of which is difficult. Botulinum toxin A (BTX-A) injection is demonstrated as efficient in treating knee adhesion after surgery. However, the treatment outcomes and the mechanism of action are not yet determined. The aim of the present study was to examine the effects and molecular mechanism of a BTX-A treatment in preventing adhesion of the knee. Twenty-four Wistar rats were randomly divided into a BTX-A treatment group and a control group. BTX-A or saline was injected into the cavity of the knee in the BTX-A treatment or control group respectively. Gross and histopathological examinations of interleukin 1 (IL-1) and fibroblast growth factor (FGF) levels, as well as fibroblast cell numbers, were assessed in the knee intra-articular adhesions in each group 6 weeks after recovery from the surgery. Macroscopic observations showed a significant reduction in adhesion severity in the BTX-A treatment group compared with the control group. In addition, the levels of IL-1 and FGF were lower and the number of fibroblasts was smaller in the BTX-A treatment group compared with those in the control group. BTX-A prevented intra-articular adhesion of knee in the rats, which might be associated with reduced expressions of IL-1 and FGF.

## Introduction

The formation of adhesion in the knee is a common complication after knee surgery [1]. The rate of arthrofibrosis following total knee arthroplasty (TKA) is estimated to be 8–12% [2] and the rate after ligament reconstruction is 0–4% [3], and it is up to 7% following a fracture of the tibial plateau high-energy fractures [4]. Arthrofibrosis of the knee leads to stiffness, a decreased range of motion and pain [5]. The loss of range of motion of the knee impairs the nutrition supply to the cartilage, resulting in cartilage degeneration and, eventually, loss of function in the knee [5]. In general, a range of motion of the knee from 0 to 125° is adequate for activities of daily living for people [6]. A loss of extension of 5° in the knee joint significantly increases the energy consumption of the quadriceps muscle and produces a limp [7].

Inhibiting the formation of the adhesions may improve the post-operative recovery of the knee [8,9]. However, as the physiological mechanisms of adhesion formation remain unclear, methods of inhibiting their formation are diverse. Various methods have been investigated, including treatment with sodium hyaluronate, chitosan, mitomycin C, fibroblast growth factor 2 (FGF-2) antibody and 10-hydroxycamptothecin, which have achieved a certain effect on the inhibition of adhesion formation [5,10–14].

Botulinum toxin A (BTX-A) is known as a neurotoxin that blocks acetylcholine release at the neuromuscular junction, causing muscle weakness, paralysis and atrophy [15,16]. It has been widely applied to treat muscle spasm, dystonia, spasticity and myofascial pain syndrome [17]. Additionally, BTX-A is also reported to improve radiation fibrosis syndrome [18]. Sahinkanat et al. [19] demonstrated that BTX-A inhibits the production of collagen during urethral wound healing. Chen et al. [20] reported that an intra-articular injection of BTX-A retards the process of fibrosis and can be used to treat frozen shoulder. These results suggest that the role of BTX-A may be associated with fibrosis and inflammation. A previous study demonstrated that an intra-articular injection of BTX-A into the rabbit knee prevented adhesion formation and reduced arthrofibrosis of the knee after knee surgery [21], but the precise mechanism remains unclear.

Therefore, in the present study, we investigated the effect of an intra-articular BTX-A injection on adhesion formation and knee arthrofibrosis after knee surgery in rats. Furthermore, some studies show that adhesion formation is reduced by inhibiting inflammation and fibrosis [22,23]. Thus, we further assessed the effect of BTX-A on the levels of inflammation/fibrosis-related cytokines and growth factors, such as interleukin 1 (IL-1) [24] and fibroblast growth factor (FGF) [25] levels, as well as the fibroblast cell numbers, in knee intra-articular adhesions.

## Materials and methods

### Animals and reagents

Twenty-four adult male Wistar rats weighing 330–380 g were used in the present study. All the rats were purchased from Jinfeng Experimental Animal Breeding Co., Ltd., Jinan (batch, SCXK 20140006). All the experimental procedures were approved by the Research Etiquette Commission (Ethics approval number: AF-SQ-04-01.0) for animal experiments. Animal care was performed according to the guidelines of the authors’ institution and the Ministry of Science and Technology of Chinese Guidance Suggestions for the Care and Use of Laboratory Animals. The animals were raised in the Animal Husbandry Facility at Qingdao University. Twenty-four rats were randomly divided into the BTX-A treatment group and the control group (*n*=12 each).

One hundred units (U) of BTX-A (Allergan Pharmaceuticals Ireland, Castlebar Road, Westport, County Mayo, Ireland; batch, S20120067) was dissolved in 1 ml saline.

### Animal model of arthrofibrosis

The method described by Kocaoglu et al. [26] was used to establish the model of arthrofibrosis. Briefly, the rats were anaesthetized with an intraperitoneal injection of 10% choral hydrate at 0.06 ml/10 g. Then, the right knee joint was shaved with electric clippers, prepped with an iodine solution and draped in aseptic conditions. After a skin incision, the knee was opened using a medial parapatellar approach and the medial and lateral sides of the femoral condyle were exposed. A partial capsulotomy and synovectomy were performed. The articular capsule and the retinaculum were closed with sutures using 3/0 non-absorbable thread. Subsequently, an intra-articular injection of 10 U (0.1 ml) BTX-A was administered to the right knee joint in the BTX-A treatment group, while the same volume of saline was given to the mice in the control group. The skin was closed with silk sutures, and the external immobilization of the surgical limb was not used post-operatively. The right leg of the rats was left free for normal movement. The rats were maintained with free access to standard chow and water. Six weeks after the operation, all the animals had recovered well without wound infection or mortality.

### Joint angle measurement

The rats were sacrificed by an intraperitoneal injection of 10% choral hydrate (0.1 ml/10 g) at 6 weeks post-surgery. The right leg of each rat was fixed, and the angle between the longitudinal axis of the femur and the tibia was measured using an angulometer with 20 g of force. The range of motion of the knee was measured three times and the mean range of motion was calculated.

### Enzyme-linked immunosorbent assay

During the surgery, the right knee of each rat was exposed and the articular capsule was kept intact. The synovial cavity of the knee joint was washed with 0.5 ml of saline, and then, the douche fluid was collected from the synovial cavity and was stored in a −20°C freezer. The levels of IL-1 and FGF in the synovial fluid were measured by ELISA according to the manufacturer’s instructions (RD Systems, Minnesota, U.S.A.) and were calculated using a Multiscan automatic enzyme mark equipment (HUMAN Gesellschaft für Biochemical und Diagnostic GmBH, Wiesbaden, Germany) using a standard curve based on the absorbance values.

### Gross observation

After irrigating the knee joint, the joint was exposed through a lateral parapatellar approach. The presence and severity of osteo-capsular adhesion was assessed using a visual scoring system (VSS) [27] by an experienced pathologist. This pathologist was specialized in joint disease and was blinded to design of the study. The degree of adhesion formation was scored as follows: 0, no adhesions; 1, weak, mild adhesions, in which the adhesion membrane was thin and could be stripped by minimal manual traction; 2, moderate adhesions that could be removed by manual traction and 3, a dense adhesion layer that could only be eliminated by surgery.

### Histological evaluation

After grading the knee intra-articular adhesions, the knee joints, including all the connective tissue and fibrotic adhesions, were excised. The samples were fixed with 10% paraformaldehyde for 48 h, decalcified with EDTA for 2 weeks and embedded in paraffin. Transverse sections perpendicular to the femoral axis were stained with haematoxylin–eosin (H&E, Chiansun Specialty Products, Co., Ltd., China). Intra-articular scar adhesions were evaluated under a light microscope at 100 magnification (Olympus microscope, CX41; Olympus, Tokyo, Japan). Three areas in the scar tissue near the bottom of the decorticated areas were selected, and fibroblast counts were performed per square under a microscope at 400× magnification.

### Statistical analysis

All the data were analysed using SPSS 15.0 software (SPSS Inc., Chicago, IL, U.S.A.) and are expressed as the mean ± S.D. A comparison of the range of motion of the knee, the number of fibroblasts and the levels of IL-1 and FGF between the BTX-A treatment and control groups were performed by a *t* test. The adhesion scores between the BTX-A treatment and control groups were compared using a Wilcoxon two-sample test with a 95% confidence level. A *P* value <0.05 was considered statistically different.

## Results

### BTX-A treatment increased the range of motion and reduced IL-1 and FGF levels

Compared with the control group, the range of motion in the joint angle measurement significantly increased in the BTX-A treatment group (Table 1). In addition, the ELISA analysis revealed that the IL-1 and FGF contents were remarkably lower in the BTX-A treatment group than in the control group (Table 2).

**Table 1:** Range of motion of the knee with scar tissue

Group	Sample	Range of motion (degree)
BTX-A treatment group	12	98 ± 13.65
Control group	12	79.42 ± 10.76
*t* value		3.70
*P value*		0.001

**Table 2:** IL-1 and FGF levels in the douche fluid (ng/l, mean ± S.D.)

Group	Sample	IL-1	FGF
BTX-A treatment group	12	2.25 ± 0.67	36.29 ± 3.17
Control group	12	3.61 ± 1.04	39.71 ± 2.54
*t* value		−3.80	−2.91
*P* value		0.001	0.008

### BTX-A treatment decreased the adhesion scores

Based on an anatomical dissection and a visual assessment under an unaided eye, the right knee joint showed mild and loose adhesions in the BTX-A treatment group, while pyknotic tubular fibrosis and arthrosclerosis were observed in the right knee joint of the rats in the control group. The VSS assay revealed that seven rats with a score of 1 and five rats with a score of 0 were found in the BTX-A treatment group; however, the control group had three rats with a score of 3, six rats with a score of 2 and three rats with a score of 1. The mean of adhesion scores was markedly lower in the BTX-A treatment group than in the control group (Table 3).

**Table 3:** Adhesion scores from the macroscopic visual scoring system

Animal	BTX-A treatment group	Control group
1	1	2
2	0	2
3	1	1
4	0	1
5	1	2
6	1	3
7	1	2
8	1	1
9	0	2
10	0	3
11	1	3
12	0	2

Mean ± S.D. values: BTX-A treatment group, 0.58 ± 0.51; Control group, 2.0 ± 0.73 (*P*<0.001).

**Table 4:** The number of fibroblasts in the scar tissue

Group	Sample	Number of fibroblasts
BTX-A treatment group	12	15.83 ± 4.53
Control group	12	28.67 ± 5.74
*t* value		–6.08
*P value*		<0.001

### Histopathological observations

**Figure 1:**
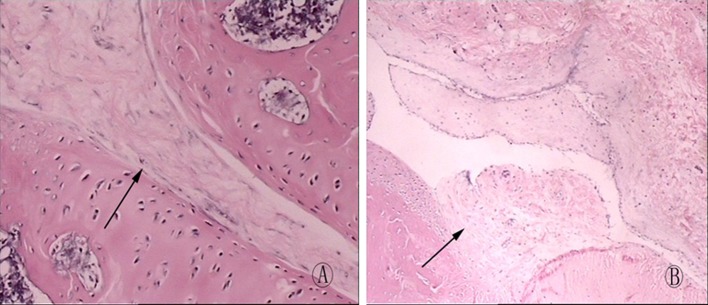
Knee joint histopathology of the BTX-A group and the control group (×400 magnitude) (**A**) The control group showed a large amount of thick and pyknotic scar formation (arrowhead) in the decorticated areas that adhered to the bone; the cavity of the knee was filled with scar tissue. There was a large amount of fibroblasts in the scar tissue. (**B**) The knee joint histopathology showed loose and thin collagenous fibre formation (arrowhead) without pyknotic adhesions in the decorticated area in the BTX-A treatment group. There were a sparse number of fibroblasts in the scar.

The H&E staining results showed that the rats in the BTX-A treatment group exhibited a moderate degree of intra-articular fibrous adhesion and had a well-organized loose pattern of collagen fibres (Figure 1A). The rats in the control group showed a large amount of pyknotic scar formation and had a poorly organized dense pattern of collagen fibres, and the scar tissue was firmly adhered to the cartilage and was accompanied by a large number of fibroblasts (Figure 1B). In addition, the quantified fibroblast numbers in the rats in the BTX-A group were significantly less than in the control group ( Tables 1 & 4).

## Discussion

Intra-articular adhesion leads to joint fibrosis and arthrosclerosis, reduces the range of knee joint movement and even causes disability [28]. Although the pathophysiological mechanism of the formation of knee intra-articular adhesion is not fully understood, fibroblastic activity and an inflammatory response are reported to be involved in the development of intra-articular adhesions [29,30]. Haematoma development, occurring after knee surgery or trauma, might lead to inflammatory cell infiltration and the production of inflammatory factors, which induces fibroblast proliferation and eventually causes adhesion scar formation [31]. In addition, immobilization and pain after knee surgery might influence the motion of the knee by inducing contracture of the tissue around the knee and the degeneration of cartilage, all of which, aggravate adhesion formation [13,31].

The present study revealed that the BTX-A treatment induced an increased range of motion, decreased adhesion and reduced the number of fibroblasts in the right knee joint of rats with arthrofibrosis. BTX-A paralyses the local muscles by inhibiting the release of acetylcholine at the neuromuscular junction [32]. It had been used for the treatment of keloids [33] and the mechanism of BTX-A, in this treatment, is thought to occur by reducing the skin tension that is caused by muscle contraction, which thereby decreases the scar tension and the subsequent inflammation at the wound edges [34]. A previous study reported that BTX-A inhibits the proliferation of fibroblasts and the synthesis of collagen [35]. BTX-A is also reported to reduce the expression of transforming growth factor (TGF)-β protein in fibroblasts derived from scars and significantly decreases their proliferation rate [36]. In addition, BTX-A has a potential analgesic effect by relaxing the muscle and blocking the pathway of pain conduction, and the pain relief might facilitate the rehabilitation process and decrease the formation of knee adhesions [37]. On the other hand, the paralysis caused by BTX-A to the muscles might actually cause knee joint degeneration. Although, when the long-term effects of intra-articular BTX-A injections to treat chronic joint pain were evaluated, the injections were found to be an effective and safe treatment for chronic joint pain disorders [38]. At any rate, such concerns are important to keep in mind while evaluating the effects of BTX-A.

All the studies mentioned above suggest that BTX-A might be associated with inflammation and fibroblast proliferation. Consistent with our results, Namazi et al. [39] reported an inhibitory effect of BTX-A on adhesion formation in the knees of rabbits. Furthermore, the present study showed that BTX-A treatment inhibited the expressions of IL-1 and FGF in the synovial fluid of the right knee joint of rats with arthrofibrosis. IL-1 is a major inflammatory factor with an extensive biological activity. IL-1 is considered a potent inflammatory cytokine that induces cartilage and bone damage and is closely associated with the pathological changes of adhesion formation [40]. IL-1 also plays an important role in stimulating metalloproteases [41], promoting pro-fibrotic mediators [42] and stimulating fibroblast proliferation and chemotaxis [43]. All of these aspects of IL-1 make it a likely player in adhesion formation.

Furthermore, FGF is an important cytokine involved in tissue repair and fibrosis formation, and elevated levels of FGF are closely related to tissue hyperplasia and scar formation [44,45]. A previous study had demonstrated that FGF stimulates the proliferation of fibroblasts and induces the synthesis of plasminogen and collagen by adjusting the proliferation and migration of endothelial cells [46]. An antibody against FGF-2, a member of the FGF family, was administered continuously for 4 weeks in the knee joint of rabbits, and the results showed that the angles of the flexion contracture and the total collagen content of the adhesion tissue were lower in the FGF-2 antibody administration group compared with the control group. In addition, the gross observation showed numerous firm adhesions on both sides of the condyle in the control group [47]. The present results indicated that the anti-adhesion effects of BTX-A might be associated with the decreased IL-1 and FGF levels in rats with arthrofibrosis. The further mechanism of the effects should be investigated by exploring for the functional role of IL-1 and FGF after BTX-A treatment *in vitro* as well as the molecular pathways involved.

In addition, the assessment of our results should also be taken into consideration that we used an intra-articular injection of BTX-A. Many studies have used intramuscular injections of BTX-A to decrease pain and increase muscle function, and the mechanism of such action include a muscle relaxant effect and the inhibition of the release of neurotransmitters by sensory neurons [48]. Such injections seem to be more appropriate for muscular pain/problems rather than for articular pain. An intra-articular injection of BTX-A was shown to be a safe and effective treatment of refractory joint shoulder pain caused by chronic arthritis [49]. The mechanism by which an intra-articular injection of BTX-A exerts this effect is not known, but it is suggested that it may involve the inhibition of the release of pain peptides from nerve terminals and sensory ganglia and anti-inflammatory and anti-glutaminergic effects [50]. Thus, there is clearly a distinction in the site of the injection that should be explored in the future.

Moreover, we used a high dose and volume of BTX-A in our model. A study by Shaari et al. [51] demonstrated that when BTX-A was injected in muscle, it spread to the surrounding muscles and this can affect its aetiology. At the dose and volume we used, we cannot say that our injection remained in the articular area and that it did not leak into the surrounding muscle. Thus, we can only assume that the muscle tissues affecting the joint must have been exposed to BTX-A. As a consequence, the muscle tissues might have become stiffer and the passive resistance of the muscles around the knee joint might have increased. Indeed, in rats injected with BTX-A in the midbelly of the tibialis anterior, the extensor digitorum longus muscle isometric forces were measured after proximal and distal lengthening, and BTX-A decreased the active muscle tone and also caused a narrower active range of movement and an increased passive resistance [52]. Furthermore, when BTX-A is injected in the midbelly of the tibialis anterior, the collagen content in the muscles exposed to BTX-A increases, and the collagen content of the connective tissues interconnecting the muscles decreases [52,53]. In the present study, we found a decreased number of fibroblasts in the cartilage of the BTX-A injected group, but based on our high dose and volume, the opposite effect of an elevated number of fibroblasts is plausible for the muscle tissue. In addition, the number of fibroblasts in the muscular connective tissues could play a role in the knee joint range of motion. These correlations are worth further exploration.

## Conclusion

BTX-A prevented the formation of intra-articular adhesion after knee surgery, which was associated with reduced IL-1 and FGF levels. Therefore, BTX-A might be considered a promising biochemical agent for controlling scar tissue formation after open and arthroscopic knee surgery.

## Abbreviations

BTX-A: botulinum toxin A
FGF: fibroblast growth factor
FGF-2: fibroblast growth factor 2
H&E: haematoxylin–eosin
IL-1: interleukin 1
TGF: transforming growth factor
VSS: visual scoring system
